# PLCγ2 controls neutrophil-like cell sensitivity through calcium oscillation and gates chemoattractant concentration range for chemotaxis

**DOI:** 10.3389/fimmu.2025.1633390

**Published:** 2025-08-01

**Authors:** Xuehua Xu, Woo Sung Kim, Arthur Lee, Tian Jin

**Affiliations:** Chemotaxis Signaling Section, Laboratory of Immunogenetics, National Institute of Allergy and Infectious Diseases, National Institutes of Health, Rockville, MD, United States

**Keywords:** chemotaxis, neutrophil sensitivity, G protein coupled receptor (GPCR), PLC gamma 2, calcium oscillation and calcium signaling, calcium promoted Ras inactivator (CAPRI)

## Abstract

The relationship between calcium oscillation and cell sensitivity is poorly understood. Calcium oscillation can occur spontaneously or be triggered upon receptor-ligand binding. The cytosolic [Ca^2+^] increase during calcium oscillation is initiated from Ca^2+^ release from the intracellular stores through the phospholipase C (PLC)-derived inositol 1,4,5-trisphosphate (IP_3_). Here, we show that neutrophil-like HL60 cells lacking PLCγ2 (*plcg2^kd^*) exhibit impaired spontaneous calcium oscillation and a diminished calcium response to chemoattractant stimulation. These defects result in reduced membrane targeting of RasGAP CAPRI (calcium-promoted Ras inactivator), and subsequent elevated Ras activations and enhanced downstream signaling, including PI_3_Kγ activation and actin polymerization. Notably, *plcg2^kd^* cells display increased sensitivity and can respond to chemoattractant gradients at a subsensitive concentrations. Taken together, our findings identify PLCγ2 as a key regulator of spontaneous and chemoattractant-induced calcium signaling and demonstrate its essential role in controlling cell sensitivity and chemoattractant concentration range for chemotaxis through CAPRI-dependent Ras signaling.

## Introduction

Calcium oscillation is ubiquitous, triggered either spontaneously or upon receptor-ligand binding in cells. They arise from coordinated intracellular Ca^2+^ release and extracellular Ca^2+^ influx. Intracellular Ca^2+^ is typically released via inositol 1,4,5-trisphosphate (IP_3_), a second messenger produced by phospholipase C (PLC), while extracellular Ca^2+^ enters through store-operated channels (SOCs) in the plasma membrane. In neutrophils, chemoattractant-induced calcium oscillation, often also called calcium response, were first documented over two decades ago (1). Genetic studies have shown that PLCβ2 and PLCβ3 are essential for this response, as their deletion markedly impairs calcium signaling in neutrophils (2). Beyond the calcium signaling, PLCβ2/β3 also regulate neutrophil polarization and chemotaxis by modulating the cofilin phosphatase slingshot-2 (3). Moreover, localized calcium pulses help coordinate lamellipodial retraction and adhesion dynamics at the leading edge (4), linking calcium signaling to cell migration (1, 5). Despite these insights, the molecular basis of spontaneous calcium oscillations remains unclear. Furthermore, the distinct roles of spontaneous versus chemoattractant-induced calcium signaling in neutrophil behavior—and particularly in modulating sensitivity to external cues—are not yet well understood.

Phospholipase C gamma (PLCγ) is a potent regulator of many signaling pathways that are essential in physiological and pathological responses of immune disorders and cancers (6). Gain-of-function mutants of PLCγ2 have been linked to severe autoimmune and immunodeficiency (7), while the consequence of PLCγ2 deficiency have drawn an increasing attention in recent years (8–11). Although several studies have suggested a potential link between altered PLC expression and Ras pathway activation, the findings remain contradictory (12–14). Neutrophils express high level of PLCγ2 in addition to PLCβ2 and PLCβ3 (15). Tyrosine phosphorylation on Y756 mediates the activation of PLCγ2 and plays an essential role in integrin/Fc receptor-mediated neutrophil functions (16). Interestingly, we have previously found that PLCγ2 is robustly recruited to the leading edge of chemotaxing neutrophils-like HL60 cells (17), while it can undergo unconventional activation via plasma membrane (PM) translocation (17–19). We further demonstrated that the chemoattractant stimulation does not induce conventional tyrosine phosphorylation at Y756 to activate PLCγ2 (20). Instead, its PM translocation and subsequent activation requires its C2-domain, indicating that C2-calcium binding mediates this process, consistent with the previous reports (18, 19). In human neutrophil-like cells with stably knocked-down of *plcg2* (*plcg2^kd^*), an altered PLC signaling, including DAG production and IP_3_-mediated calcium response demonstrates the involvement of PLCγ2 in chemoattractant-mediated PLC signaling in HL60 cells (20). Specifically, *plcg2^kd^* cells display a significantly reduced duration of calcium response upon chemoattractant stimulation at saturating concentrations of chemoattractant. Neutrophils also highly express CAPRI (Calcium-promoted Ras inactivator), a Ras GTPases-activating protein (RasGAP) containing a calcium-binding C2-domain that facilitates its translocation to plasma membrane upon cytosolic [Ca2^+^] increase (21). CAPRI serves as a key mediator linking calcium signaling to Ras activation (22). In *plcg2^kd^* HL60 cells, impaired calcium signaling leads to defective plasma membrane (PM) recruitment of CAPRI. Reduced CAPRI membrane translocation in *plcg2^kd^* cells results in elevated and prolonged Ras activation. This sustained Ras activity leads to hyperactivation of PI3K and its downstream effectors, including the PI3K–GSK3–cofilin axis. Consequently, *plcg2^kd^* cells exhibit excessive actin polymerization, impaired front–rear polarization, and defective chemotaxis (20). We have also previously shown that CAPRI regulates cell sensitivity and defines the concentration range of chemoattractant gradients for effective chemotaxis (23). Here, we investigated the role of PLCγ2 in spontaneous calcium oscillation, focusing on its effect on PM targeting of CAPRI, Ras activation, and downstream signaling in neutrophils. Our findings demonstrate that PLCγ2 not only mediates spontaneous calcium oscillation but also modulates chemoattractant-triggered calcium signaling, thereby regulating cell sensitivity and gating chemoattractant concentration ranges for chemotaxis through membrane CAPRI-dependent Ras signaling.

## Materials and methods

### Cell culture and differentiation

The culturing of control and *plcg2kd* HL60 cells was as previously reported. Briefly, cells were maintained in RPMI 1640 culture medium [RPMI 1640 medium with 20% (v/v) fetal bovine serum and 25 mM HEPES (Quality Biological, Inc. Gaithersburg, MD)]. HL60 cells were differentiated in RPMI 1640 culture medium containing 1.3% DMSO for 5 days before the experiments. The cells were incubated at 37°C in a humidified 5% CO_2_ atmosphere.

### Plasmids and transfection of cells

The DNA vectors of turboGFP (tGFP)-human CAPRI, active Ras sensor (active Ras binding domain of human Raf1 tagged with mRFP, RBD-RFP), PIP_3_ biosensor (PH-GFP), and PM markers (CAAX-mCherry) were from Addgene (Cambridge, MA). The F-actin sensor F-tractin-GFP was obtained from John Hammer (24). The transfection procedure was as previously described (25). Briefly, 2 × 10^6^ cells were centrifuged at 100 × *g* for 10 min and resuspended in a mixture of 80 µL nucleofection solution V and 20 μL supplement I at room temperature. Six micrograms of plasmid DNA encoding the cDNA of the desired proteins were used for a single transfection reaction using program T-019 on the Amaxa Nucleofector II (Lonza, MD).

### Calcium response

Cells were incubated with 100 ng/ml Fluo4 (Invitrogen, Carlsbad, CA) at 37°C for 30 min, washed with RPMI 1640 medium with 25 mM HEPES twice to remove the unstained Fluo-4, and then subjected to the experiments.

### Ras activation assay

Briefly, cells were starved in RPMI medium containing 25 mM HEPES at 37°C for 3 hours. Cells were then collected, resuspended at 2×10^7^ cells/ml, and transfer to a medical cup under constant shaking at 200 rpm for 3 min at room temperature. Cells were stimulated with fMLP at the indicated final concentrations. At the indicated time points before or after stimulation, 100 μl aliquots of the cells were taken from the shaking medical cup to ensure equal number of cells were subject to the subsequent steps. Aliquots were then mixed with immunoprecipitation buffer (IB), including 0.25% NP40, 10 mM Tris (pH7.5) buffer, 150 mM NaCl, 1 mM Na3VO4, 10 mM NaF, and 1X proteinase inhibitor (Rhoche, Basel, Switzerland). The mixtures were incubated on ice for 30 min and then centrifuged at 100,000 × *g* at 4 °C for 30 min. The supernatants were incubated with agarose beads conjugated with RBD (active Ras binding domain of human Raf1) (Cytoskeleton, Inc. Denver, CO) at 4°C for 2 hours. The agarose beads were washed three times with IB. The protein on the beads was eluted by mixing with 25 μl 2X sample loading buffer (SLD) (Quality Biological Inc, Gaithersburg, MD). The supernatants and eluted proteins were subjected to western blot detection of the indicated proteins.

### Imaging and data processing

Cells were plated and allowed to adhere to the cover glass of a 4-well or a 1-well chamber (Nalge Nunc International, Naperville, IL) precoated with Fibronectin (Sigma Aldrich, Saint Louis, MO) for 10 min, and then covered with RPMI 1640 medium with 10% FBS and 25 mM HEPES. For confocal microscopy, cells were imaged using a Carl Zeiss Laser Scanning Microscope Zen 780 (Carl Zeiss, Thornwood, NY) with a Plan-Apochromat 60x/1.4 Oil DIC M27 objective. For the uniform-stimulation experiment of membrane translocation assays, the stimuli were directly delivered to the cells as previously described (25). To visualize the application of the stimuli, Alexa 633 or Alexa 488 was mixed with the fMLP stimuli at a final concentration of 1 μg/ml. For calcium response analysis, identical imaging parameters were used for both CTL and *plcg2kd* cells. Fluo-4 fluorescence intensity was measured in individual cells before and after stimulation, and the data were extracted and analyzed using GraphPad Prism. A two-tailed unpaired student *t*-test was used to calculate the *p*-value for the comparisons of peak responses between CTL and *plcg2^kd^* cells. Statistical significance is indicated as follows: ns (not significant *p* > 0.1), *(*p* < 0.1), **(*p* < 0.01), ***(*p* < 0.001). The membrane translocation of the indicated protein was measured by the depletion of the interested protein in the cytoplasm as previously described (23). The data obtained were further analyzed with Microsoft Office Excel (Redmond, WA). For quantitative analysis of membrane translocation dynamics of the indicated molecules, the cytosolic depletion of the indicated molecule was measured. Regions of interest (ROIs) in the cytoplasm (avoiding the nucleus area as much as possible) were within the cells throughout the time period of the measurements. The periphery of the cells was marked by the membrane markers. For data analysis, to normalize the effect of photobleaching during data acquisition, the intensity of ROIs in the cytoplasm was divided by the intensity of whole cells at each given time point. To normalize the effect of morphological change during the time period, the above resulting data were divided by the intensity of ROIs in the PM marker channel in the case PM marker was simultaneously monitored. Lastly, the resulting data were divided by that at time 0 s; consequently, the relative intensity of any cells at time 0 s became 1. The graph of mean ± SD is shown.

### TAXIScan chemotaxis assay and data analysis

The procedure was as previously reported (26). Briefly, differentiated cells were loaded onto fibronectin-coated 4-µm EZ-TAXIScan chambers. The chemoattractants at the indicated concentrations were added to the other side of the well across the terrace that the cells chemotax through. The cells migrated for 30 min at 37°C. Images were taken at 30-s intervals. For chemotaxis parameter measurements, 20 cells in each group were analyzed with DIAS software (27). The bar graphs of chemotaxis parameters in mean and SD were plotted with Microsoft Office Excel (Redmond, WA).

## Results

### Impaired spontaneous calcium oscillation and chemoattractant-induced calcium response in *plcg2^kd^* cells

**Figure 1:**
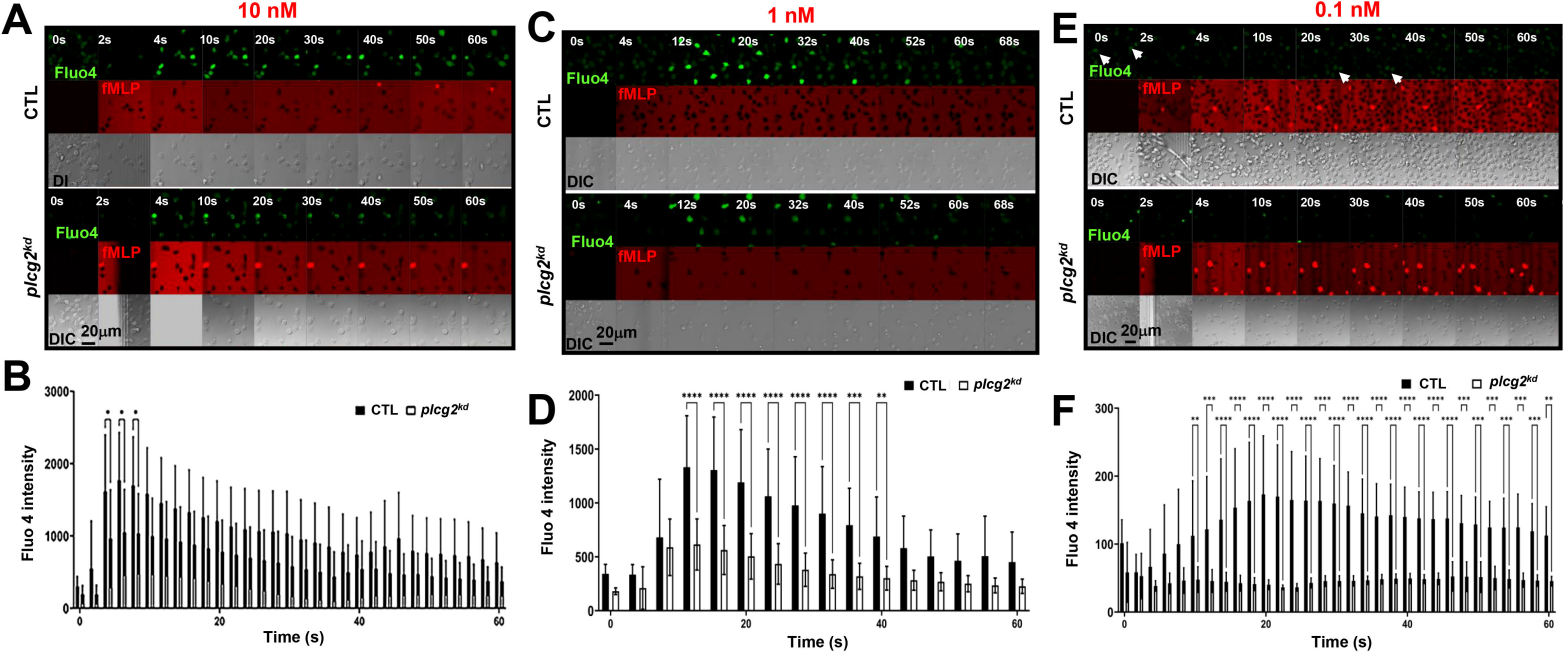
Decreased calcium response in *plcg2^kd^* cells upon fMLP stimulation. **(A)** Montages show a 10 nM fMLP-induced calcium response in control (CTL) and *plcg2*-stably knocked down (*plcg2^kd^*) cells. Cells stained with the calcium indicator, Fluo-4 (green), were stimulated with fMLP at the indicated concentrations of fMLP at time 0 s. To visualize the application of fMLP stimuli, fMLP was mixed with a fluorescent dye, Alexa 633 (red). Image acquisition conditions of CTL and *plcg2^kd^* cells are same in **(A, C, D)** Scale bar = 20 μm. For complete sets of cell responses, see **Supplementary Video S1**. CTL cells are in the upper panel, and *plcg2^kd^* cells are in the lower panel. The appearance of red indicates the application of fMLP stimulation. **(B)** Dot plot analysis of Fluo-4 intensity change in CTL and *plcg2^kd^* cells before and after 10 nM fMLP stimulation in **A** and two other independent experiments. N = 10 or 13 for CTL or *plcg2^kd^* cells, respectively. A two-tailed unpaired student *t*-test was used to calculate the *p*-value for the comparisons of peak responses between CTL and *plcg2^kd^* cells. Statistical significance is indicated as follows: ns (not significant *p* > 0.05), *(*p* < 0.05), **(*p* < 0.01), ***(*p* < 0.001). The same statistical analysis was applied in **(D, F)**. **(C)** Montages show 1 nM fMLP-induced calcium response in CTL and *plcg2^kd^* cells. Cells stained with Fluo-4 (green) were stimulated with 1 nM fMLP at time 0 s. To visualize the application of fMLP stimuli, fMLP was mixed with a fluorescent dye, Alexa 633 (red). Scale bar = 20 μm. For complete sets of cell responses, see **Supplementary Video S2**. CTL cells are in the upper panel, and *plcg2^kd^* cells are in the lower panel. The appearance of red indicates the application of fMLP stimulation. **(D)** Dot plot analysis of Fluo-4 intensity change in CTL and *plcg2^kd^* cells before and after 1 nM fMLP stimulation in **C** and the other two independent experiments. N = 14 or 14 for CTL or *plcg2^kd^* cells, respectively. **(E)** Montages show 0.1 nM fMLP-induced calcium response in CTL and *plcg2^kd^* cells. For complete sets of cell responses, see **Supplementary Video S3**. CTL cells are in the upper panel, and *plcg2^kd^* cells are in the lower panel. The appearance of red indicates the application of fMLP stimulation. Arrows highlight individual cells that display spontaneous, asynchronous calcium oscillations. **(F)** Dot plot analysis of Fluo-4 intensity change in CTL and *plcg2^kd^* cells before and after 0.1 nM fMLP stimulation in **E** and two other independent experiments. N = 11 or 12 for CTL or *plcg2^kd^* cells from multiple independent experiments, respectively.

Calcium oscillations are a ubiquitous signaling phenomenon that occurs spontaneously or are triggered by receptor-ligand binding. In this study, we distinguish these two mechanisms by referring to the non-ligand-induced [Ca^2+^] increase as spontaneous calcium oscillation (or calcium oscillation), and the ligand-induced one as calcium response. We previously reported fMLP-induced calcium responses in control (CTL) HL60 cells and *plcg2* stably knocked-down (*plcg2^kd^*) HL60 cells (20). Upon saturating fMLP stimulation, both CTL and *plcg2^kd^* cells exhibited comparable peak calcium. However, *plcg2^kd^* cells displayed a significantly shorter duration of calcium elevation and a marked reduction in secondary sporadic calcium spikes, suggesting that PLCγ2 plays a role in sustaining calcium signaling. To further dissect the function of PLCγ2 in calcium response and spontaneous calcium oscillation, we monitored calcium dynamics in both CTL and *plcg2^kd^* cells upon stimulation with three concentrations of fMLP (10 nM, 1 nM, and 0.1 nM). *plcg2^kd^* cells exhibit approximately 90% knockdown efficiency of PLCγ2, as previously shown (20). To visualize stimulus application, especially at subsensitive concentrations, we co-applied fMLP with Alexa 633 (red) and monitored calcium signaling using the Fluo-4 indicator (green) (**Figure 1**). At 10 nM and 1 nM fMLP, both CTL and *plcg2kd* cells exhibited calcium responses (**Figures 1A, C**, **Supplementary Video S1, S2**). CTL cells typically show the initial synchronized calcium rise—likely corresponding to the chemoattractant-induced response—and the subsequent sporadic, asynchronous calcium oscillation observed in individual cells. However, the amplitude and duration of calcium signals were significantly reduced in *plcg2^kd^* cells (**Figures 1B, D**). Calcium response in each individual CTL and *plcg2^kd^* cell further confirms the above observation (**Supplementary Figure S1**). indicating that PLCγ2 contributes to both the amplitude and persistence of GPCR-mediated calcium responses upon stimuli at a moderate ligand concentration. Upon 0.1 nM fMLP stimulation (a subsensitive concentration for CTL cells in the previous report) (23), neither cell type show synchronized calcium response (**Figure 1E**, **Supplementary Video S3**) (23). Importantly, CTL cells display sporadic calcium responses and spontaneous calcium oscillation as previously described (1). In contrast, *plcg2^kd^* cells rarely showed sporadic calcium activity (a representative responses of multiple *plcg2kd* cells shown in **Figure 1E**, lower panel), indicating a critical role of PLCγ2 in maintaining spontaneous calcium oscillation under resting conditions. Together, these findings demonstrate that PLCγ2 not only constitutes the GPCR-mediated calcium response but also mediates the spontaneous calcium oscillation in the resting neutrophils.

### Reduced membrane translocation of CAPRI in *plcg2^kd^* cells upon fMLP stimulations

**Figure 2:**
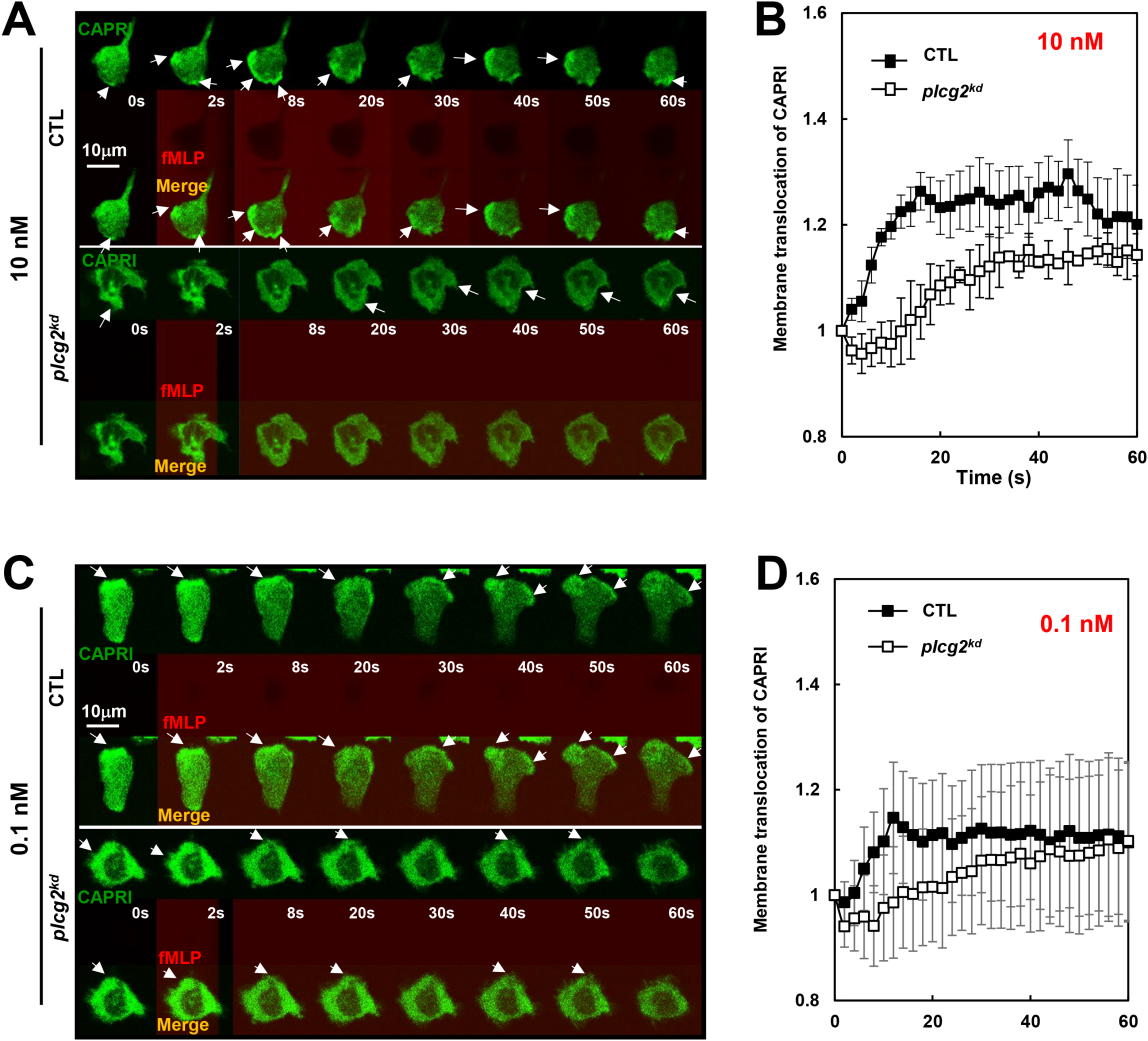
Reduced membrane translocation of CAPRI in *plcg2^kd^* cells upon fMLP stimulations. **(A)** Montages show fMLP-induced plasma membrane (PM) translocation of CAPRI-GFP in CTL and *plcg2^kd^* cells in response to fMLP stimulation at 10 nM. Cells expressing CAPRI-GFP (green) were stimulated with fMLP stimulation at 10 nM at time 0 s. To visualize the application of the stimuli, fMLP was mixed with fluorescent dye, Alexa 633 (red). Scale bar = 10 μm. Arrows indicate the localization of CAPRI-GFP before or after stimulation. See **Supplementary Video S4** for complete sets of cell responses. The CTL cell is in the upper panel, and the *plcg2^kd^* cell is in the lower panel. **(B)** Quantitative measurement of the membrane translocation of CAPRI-GFP in CTL and *plcg2^kd^* cells in response to 10 nM fMLP stimulation. To assess CAPRI-GFP membrane translocation quantitatively, cytosolic depletion of CAPRI-GFP was measured as previously reported (20). Regions of interest (ROIs) were selected in the cytoplasm, avoiding the nuclear area whenever possible, and were tracked throughout the imaging period. Due to the migratory behavior of the cells, 5 CTL and *plcg2^kd^* that exhibited typical responses with minimal movement were selected for quantitative analysis. Mean ± SD is shown; n = 5 or 5 for CTL or *plcg2^kd^* cells, respectively. **(C)** Montages show fMLP-induced plasma membrane (PM) translocation of CAPRI-GFP in CTL and *plcg2^kd^* cells in response to fMLP stimulation at 0.1 nM. Cells expressing CAPRI-GFP (green) were stimulated with fMLP stimulation at 0.1 nM at time 0 s. To visualize the application of the stimuli, fMLP was mixed with fluorescent dye, Alexa 633 (red). Arrows indicate the localization of CAPRI-GFP before or after stimulation. Scale bar = 10 μm. See **Supplementary Video S5** for a complete set of cell responses. CTL cell is in the upper panel and *plcg2^kd^* cell is in the lower panels. **(D)** Quantitative measurement of the membrane translocation of CAPRI-GFP in CTL and *plcg2^kd^* cells in response to 0.1 nM fMLP stimulation. The same quantification method used in **Figure 1B** was applied, measuring cytosolic depletion of CAPRI-GFP to assess membrane translocation. Mean ± SD is shown; n = 5 or 5 for CTL or *plcg2^kd^* cells, respectively.

The direct connection between calcium oscillation and cell sensitivity to chemoattractant stimulation is unclear. We have previously demonstrated that CAPRI mediates the deactivation of the GPCR-mediated Ras signaling to facilitate Ras adaptation in human neutrophils (23). In resting neutrophils, CAPRI is predominantly cytosolic; however, a fraction of CAPRI localizes in the plasma membrane (PM), where it regulates basal Ras activity and thereby modulate cell sensitivity. The PM localization of CAPRI depends on its C2-domain and a proper increase in intracellular calcium ([Ca^2+^]). Nonetheless, whether PLCγ2 contributes to the calcium increase required for CAPRI membrane recruitment in resting cells has not been fully elucidated. As previously reported (23), migrating CTL cells actively recruit CAPRI-GFP to the leading fronts, whileas *plcg2^kd^* cells rarely show CAPRI-GFP enrichment at the protrusion sites (**Figure 2A**). Upon 10 nM fMLP stimulation, CTL cells display a robust membrane translocation of CAPRI-GFP (**Figure 2A**, upper panel, and **Supplementary Video S4**, upper panel). In contrast, *plcg2^kd^* cells display significantly reduced PM translocation of CAPRI-GFP (**Supplementary Video S4**, lower panel), consistent with the previous report. Upon 0.1 nM fMLP stimulation, neither CTL nor *plcg2^kd^* cells show detectable CAPRI-GFP membrane translocation (**Figure 2C**, **Supplementary Video S5**). While migrating CTL cells consistently localize CAPRI-GFP to the leading fronts, while *plcg2^kd^* cells do not. We confirmed the above observation in many cells and proceeded to quantify CAPRI plasma membrane (PM) translocation in both CTL and *plcg2^kd^* cells. To assess membrane translocation quantitatively, we measured cytosolic depletion of CAPRI-GFP as previously reported. Regions of interest (ROIs) were selected in the cytoplasm, avoiding the nucleus area whenever possible, and were tracked throughout imaging period. Due to the migratory behavior of the cells, most of the cells were not suitable for quantitative measurement over time. Therefore, we selected 4 to 5 cells that displayed typical cell response with minimal movement for quantitative analysis. The results shown in **Figure 2B** and **2D** support our observations. Collectively, these results indicate that membrane targeting of CAPRI is impaired in *plcg2^kd^* cells under both resting and stimulated conditions.

### Increased Ras activation in *plcg2^kd^* cells in response to fMLP stimulation at a low or a subsensitive concentration

**Figure 3:**
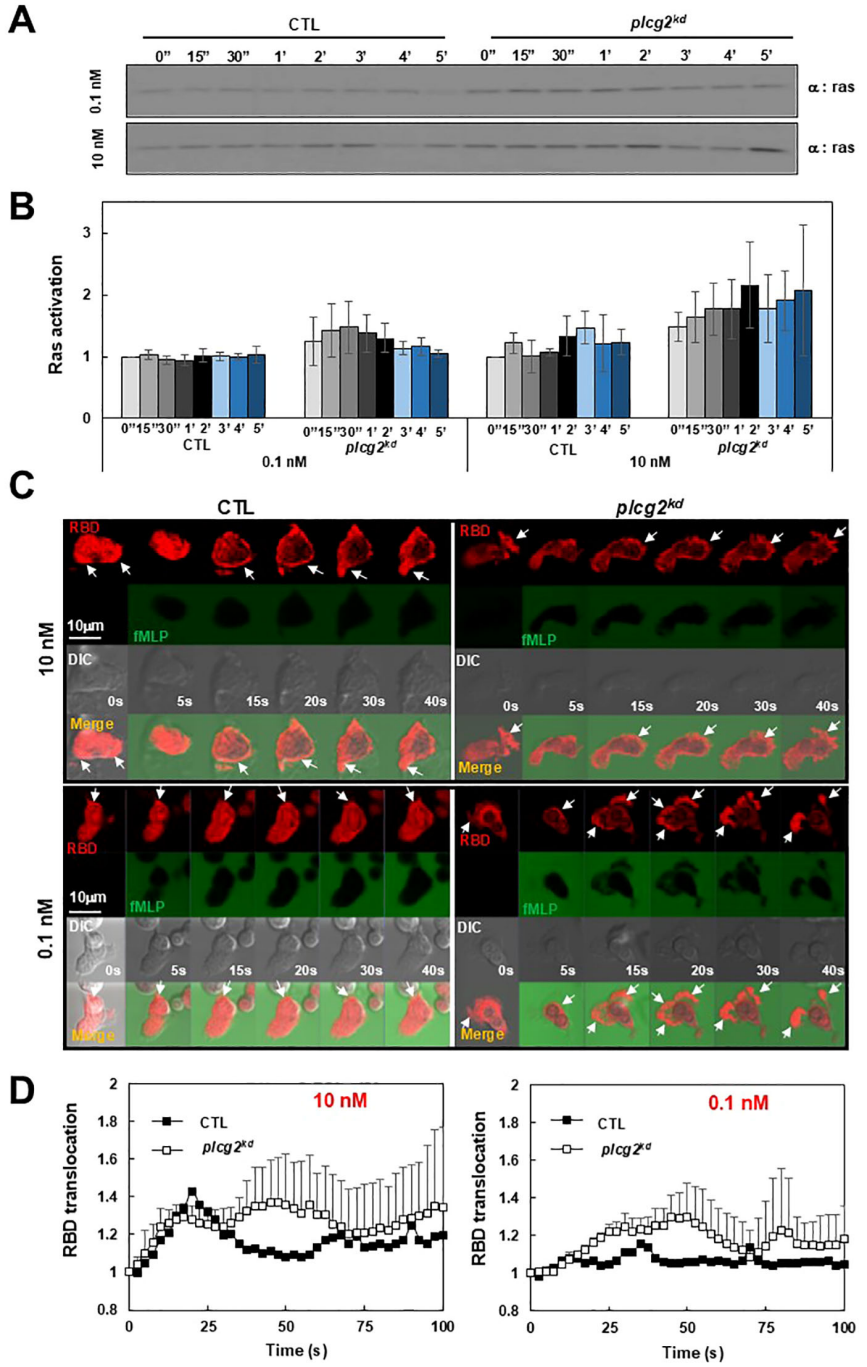
Increased Ras activation in *plcg2^kd^* cells in response to fMLP stimulation at a low (10 nM) or a subsensitive (0.1 nM) concentration of fMLP. **(A)** Ras activation in CTL and *plcg2^kd^* cells in response to either 10 nM or 0.1 nM fMLP stimulation was determined by a pull-down assay. **(B)** Normalized quantitative densitometry of the active Ras from three independent experiments, including the result presented in **(A)** The other time points are the ratio of intensity at the given time point (I_t_) vs intensity at time 0 (I_0_) and the intensity of active Ras in CTL cells at time 0 s was normalized to 1. Mean ± SD from the three independent experiments is shown. **(C)** Montage shows fMLP-induced Ras activation in CTL (left) and *plcg2^kd^* (right) cells by the membrane translocation of the active Ras biosensor RBD-RFP. Cells expressing RBD-RFP (red) were stimulated with 10 nM (upper panel) or 0.1 nM (lower panel) fMLP at time 0 s. To visualize the application of fMLP, it was mixed with a fluorescent dye, Alexa 488 (green). Arrows indicate the localization of RBD-RFP before or after stimulation. Scale bar = 10 μm. See **Supplementary Video S6 or S7** (CTL, left panel; *plcg2^kd^*, right panel) for complete sets of cell responses upon fMLP stimulation at 10 nM or 0.1 nM, respectively. **(D)** Quantitative measurement of PM translocation of RBD-RFP in CTL and *plcg2^kd^* cells in response to fMLP stimulation at either 10 nM (left) or 0.1 nM (right). Mean ± SD is shown. N = 4 or 4 for CTL or *plcg2^kd^* cells, respectively, in both graphs.

To investigate the consequence of impaired PM targeting of CAPRI in *plcg2^kd^* cells, we biochemically assessed Ras activation using a pull-down assay in a large population of both CTL and *plcg2^kd^* cells upon fMLP stimulation at different concentrations as previously reported (23). Same amount of CTL and plcg2kd cells were collected and basolated on ice for 10 min. Stimulation at a final concentration of 10 nM fMLP triggered a clear Ras activation in CTL cells, with a significantly stronger activation in *plcg2^kd^* cells (**Figure 3A**). In contrast, 0.1 nM fMLP did not trigger notable Ras activation in CTL cells but induced a clear Ras activation in *plcg2^kd^* cells, indicating increased sensitivity in the cells lacking PLCγ2. Densitometric analysis from three independent experiments (**Figure 3B**) supports this observation. The intensity of active Ras in CTL cells at time 0 s was normalized to 1, and values at other time points are presented as the ratio of intensity at the given time point (I_t_) to that at time 0 (I_0_). To further confirm the increased sensitivity of *plcg2^kd^* cells, we monitored the temporospatial Ras activation using a live-cell confocal imaging with the active Ras probe (RBD-RFP, red) (**Figure 3C**). fMLP was co-applied with Alexa488 (green) to visualize the stimulus application. RBD-RFP was found to localize at the protrusion site of both resting CTL and *plcg2^kd^* cells (**Supplementary Video S6**). Upon 10 nM fMLP stimulation, CTL cells exhibited a robust PM translocation, *plcg2^kd^* cells showed a significantly stronger and more sustained PM translocation of RBD-RFP, consistent with the previous report (**Figure 3C**, upper panel, **Supplementary Video S6**) (20). When stimulated with 0.1 nM fMLP, CTL cells did not display a marked overall PM translocation of RBD-RFP; instead, RBD-RFP remained localized to the protrusion site or the leading front of a migrating cell (**Figure 3C**, lower panel, **Supplementary Video S7**, upper panel). In contrast, *plcg2^kd^* cells showed a clear PM translocation of RBD-RFP followed by sustained localization at expanding protrusion sites upon the same low-dose fMLP stimulation (**Supplementary Video S7**, lower panel). Quantitative measurement of RBD-RFP membrane translocation across multiple cells further confirms the above observation (**Figure 3D**). In summary, *plcg2kd* cells exhibit enhanced Ras activation upon chemoattractant stimulation and respond to concentrations of fMLP that are subsensitive for CTL cells. These findings demonstrate that PLCγ2 is not only required for chemoattractant-induced Ras adaptation but also plays a critical role in regulating neutrophil sensitivity through CAPRI membrane recruitment.

### Increased PI_3_K activation in *plcg2^kd^* cells in response to fMLP stimulation at a low or a subsensitive concentration

**Figure 4:**
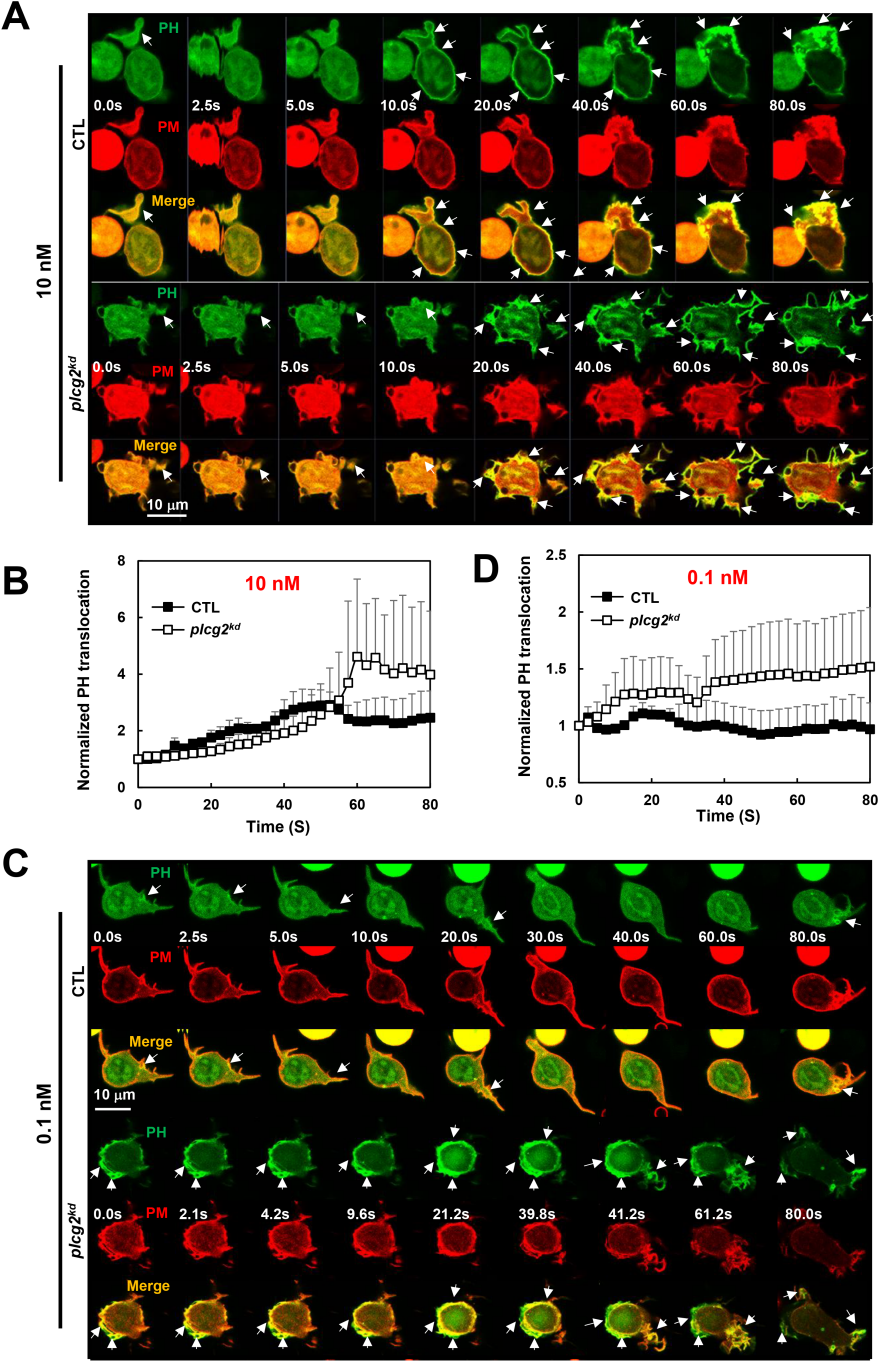
Increased PI_3_K activation in *plcg2^kd^* cells in response to fMLP stimulation at a low (10 nM) or a subsensitive (0.1 nM) concentration. **(A)** Montage shows PI_3_K activation in CTL and *plcg2^kd^* cells in response to 10 nM fMLP stimulation by monitoring PIP_3_ production using fluorescent microscopy. PIP_3_ production is visualized by the membrane translocation of the PIP_3_ biosensor PH-GFP. Cells expressing PH-GFP (green) and a PM marker (red) were stimulated with 10 nM fMLP at time 0 s. Arrows indicate the localization of PH-GFP before or after stimulation. Scale bar = 10 μm. See **Supplementary Video S8** (CTL, upper panel; *plcg2^kd^*, lower panel) for complete sets of cell responses upon 10 nM fMLP, respectively. **(B)** Quantitative measurement of PIP_3_ production by the membrane translocation of PH-GFP in CTL and *plcg2^kd^* cells in response to 10 nM fMLP stimulation. Mean ± SD is shown; n = 3 or 5 for CTL or *plcg2^kd^* cells, respectively. **(C)** Montage shows PI_3_K activation in CTL and *plcg2^kd^* cells in response to 0.1 nM fMLP stimulation by monitoring PIP_3_ production using fluorescent microscopy. Cells expressing PH-GFP (green) and a PM marker (red) were stimulated with 0.1 nM fMLP at time 0 s. Arrows indicate the localization of PH-GFP before or after stimulation. Scale bar = 10 μm. See **Supplementary Video S9** (CTL, upper panel; *plcg2^kd^*, lower panel) for complete sets of cell responses upon 0.1 nM fMLP stimulation. **(D)** Quantitative measurement of PIP_3_ production by the membrane translocation of PH-GFP in CTL and *plcg2^kd^* cells in response to 10 nM fMLP stimulation. Mean ± SD is shown; n = 5 or 5 for CTL or *plcg2^kd^* cells, respectively.

PI_3_Kγ, a direct effector of Ras, catalyzes the conversion of phosphatidylinositol (4,5)-bisphosphate (PI(4,5)P2, PIP_2_) to phosphatidylinositol (3,4,5)-trisphosphate (PtdIns(3,4,5)P3, PIP_3_) (2, 28, 29). PI_3_Kγ activation recruits and activates the PIP_3_-binding serine/threonine kinase Akt on the plasma membrane, which plays a critical role in neutrophil chemotaxis (3). To examine the consequences of the increased Ras activation in *plcg2^kd^* cells, we next investigated PI_3_K activation in both CTL and *plcg2^kd^* cells. PI_3_K activation was monitored by visualizing PIP_3_ production using a PIP_3_ biosensor, PH-GFP, through confocal microscopy. In the resting cells, PH-GFP (green) localizes predominantly in the cytosol (**Figure 4A**). It also colocalizes with a plasma membrane (PM) marker (red) at the protrusion sites as previous report (17), which appeared more pronounced in *plcg2^kd^* cells. Upon 10 nM fMLP stimulation, CTL cells displayed a transient translocation of PH-GFP to the cell periphery, followed by sustained colocalization with the PM marker at protruding sites (**Figure 4A**, upper panel, **Supplementary Video S8**). Upon the same stimulation, *plcg2^kd^* cell shows a significantly stronger and more prolonged PH-GFP translocation to PM (**Figure 4A**, lower panel; **Supplementary Video S8**). When stimulated with 0.1 nM fMLP, CTL cells did not show a clear global PM translocation of PH-GFP; instead, PH-GFP remained localized at the protrusion site of migrating cells (**Figure 4C**, upper panel, **Supplementary Video S9**). In contrast, *plcg2^kd^* cells exhibited a clear PM translocation of PH-GFP, followed by continuous localization in the expanding protrusion sites upon the same 0.1 nM fMLP stimulation, (**Figure 4C**, lower panel, **Supplementary Video S9**, **Figure 4D**). We confirmed the above observation in many cells and proceeded to quantify PH-GFP PM translocation in both CTL and *plcg2^kd^* cells. Cytosolic depletion of PH-GFP was measured using the same approach. ROIs in the cytoplasm were selected and tracked over time. To correct for photobleaching, cytoplasmic ROI intensity was normalized to the whole-cell intensity at each time point. To account for morphological changes, the values were further normalized to the intensity of the PM marker channel. Finally, all data were normalized to the intensity at time 0 s, so that the relative intensity at time 0 was set to 1. This quantitative analysis supports our observations (**Figures 4B, D**). *plcg2^kd^* cells display a significantly stronger PM translocation of PH-GFP (**Supplementary Figure S2**). In conclusion, *plcg2kd* cells exhibit elevated PI3K activation and are capable of responding to chemoattractant stimulation at concentrations that are subsensitive for CTL cells.

### Increased actin polymerization in *plcg2^kd^* cells in response to fMLP stimulation at a low or a subsensitive concentration

**Figure 5:**
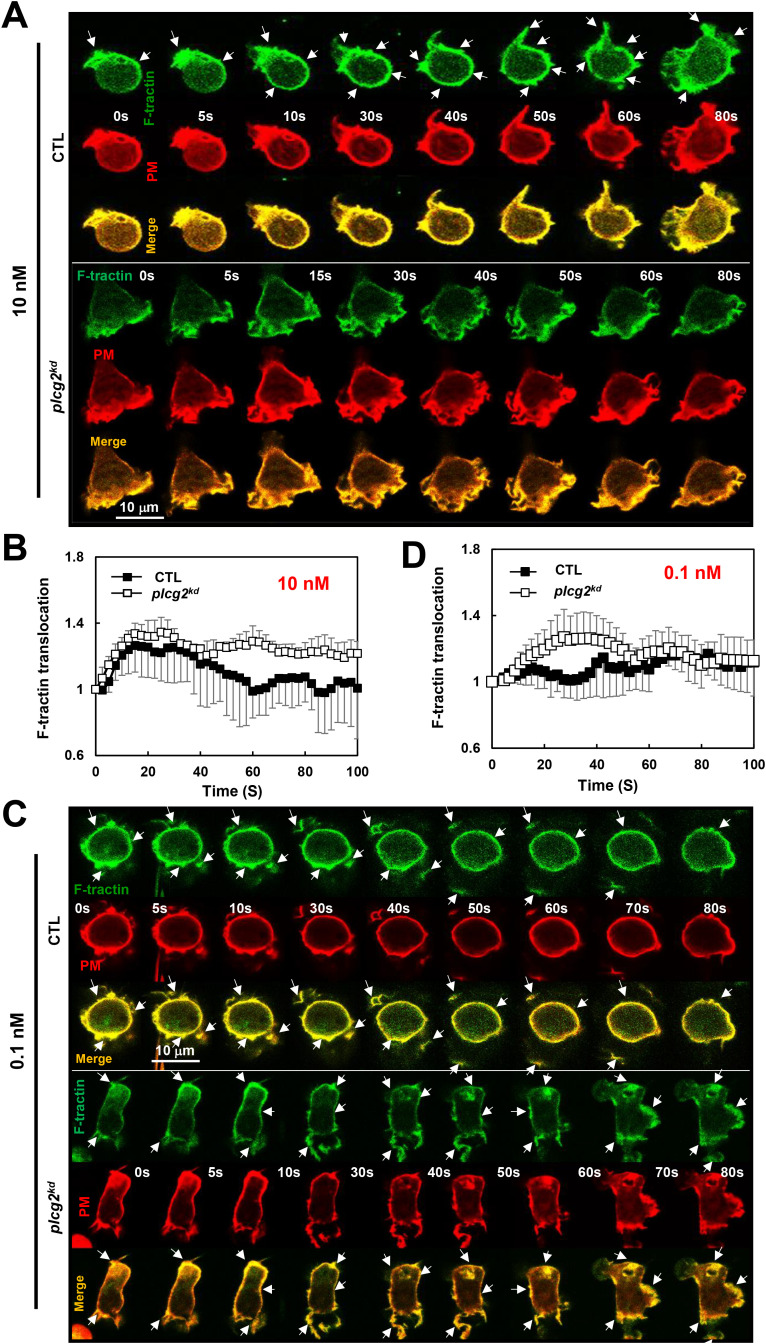
Increased actin polymerization in *plcg2^kd^* cells in response to fMLP stimulation at a low (10 nM) or a subsensitive (0.1 nM) concentration. **(A)** Montage shows the membrane translocation of the F-actin probe (GFP-tagged F-tractin) in CTL and *plcg2^kd^* cells upon fMLP stimulation at 10 nM. Cells expressing F-tractin GFP (green) and a PM marker (red) were stimulated with fMLP at time 0 s. Scale bar = 10 μm. Arrows indicate the localization of F-tractin before or after stimulation. See **Supplementary Video S10** (CTL, upper panel; *plcg2^kd^*, lower panel) for a complete set of cell responses upon 10 nM fMLP stimulation. **(B)** Quantitative measurement of actin polymerization by the membrane translocation of Ftractin-GFP in CTL and *plcg2^kd^* cells in response to fMLP stimulation. Mean ± SD is shown; n = 3 or 5 for CTL or *plcg2^kd^* cells, respectively. **(C)** Montage shows the membrane translocation of the F-actin probe (GFP-tagged F-tractin) in CTL and *plcg2^kd^* cells upon fMLP stimulation at 0.1 nM. Cells expressing F-tractin GFP (green) and a PM marker (red) were stimulated with fMLP at time 0 s. Scale bar = 10 μm. Arrows indicate the localization of F-tractin before or after stimulation. See **Supplementary Video S11** (CTL, upper panel; *plcg2^kd^*, lower panel) for a complete set of cell responses upon 0.1 nM fMLP stimulation. **(D)** Quantitative measurement of actin polymerization by the membrane translocation of Ftractin-GFP in CTL and *plcg2^kd^* cells in response to 0.1 nM fMLP stimulation. Mean ± SD is shown; n = 3 or 5 for CTL or *plcg2^kd^* cells, respectively.

Neutrophils utilize GPCR/G protein complexes to regulate multiple signaling pathways that coordinate actin cytoskeleton dynamics and drive cell migration. To evaluate the role of PLCγ2 in chemoattractant GPCR-mediated actin assembly in neutrophils, we monitored actin polymerization using a fluorescent F-actin probe, F-tractin–GFP (green), in live cells via fluorescence microscopy (**Figure 5**). In resting cells, F-tractin-GFP (green) localizes primarily in the cytosol and cortical regions, where it colocalizes with a plasma membrane (PM) marker (red) on the membrane and protrusion sites (**Figure 5A**). Upon 10 nM fMLP stimulation at 2 s, more F-tractin-GFP translocated to the cell cortex at around 10 to 40 s, then mostly returned to the cytosol at about 60 s, and then translocated to the leading front again at around 80 s in CTL cells (**Figure 5A**, upper panel, **Supplementary Video S10**). In response to the same 10 nM fMLP stimulation, *plcg2^kd^* cells displayed a continuous, persistent translocation of F-tractin-GFP and colocalized with the PM marker on the plasma membrane (**Figure 5A**, lower panel, **Supplementary Video S10**). Using the same quantification method as in **Figure 4B**, we further quantified the actin polarization of CTL and *plcg2^kd^* cells by the membrane translocation of F-tractin-GFP and confirmed that *plcg2^kd^* cells display elevated and prolonged actin polymerization compared to CTL cells (**Figure 5B**). We further determined the actin polymerization of both CTL and *plcg2^kd^* cells to 0.1 nM fMLP stimulation. In response to 0.1-nM fMLP stimulation, most CTL cells (∼90%) did not show the clear membrane translocation of F-tractin–GFP to the PM, while they showed cortex localization of F-tractin and PM marker (**Figure 5C**, upper panel, **Supplementary Video S11**). In contrast, more than 80% of *plcg2^kd^* cells showed the clear membrane translocation of F-tractin-GFP upon 0.1 nM fMLP stimulation (**Figure 5C**, lower panel, **Supplementary Video S11**). Quantitative measurement of membrane translocation of F-tractin in CTL and *plcg2^kd^* cells shows a normal oscillation of actin polymerization in CTL cells, while a clear actin polymerization in *plcg2^kd^* cells upon 0.1 nM fMLP stimulation (**Figure 5D**). Together, these results demonstrate that *plcg2kd* cells are capable of initiating actin polymerization in response to chemoattractant stimulation at subsensitive concentrations and exhibit prolonged, elevated actin assembly in response to low-dose chemoattractant stimulation.

### *plcg2^kd^* neutrophils chemotax in chemoattractant gradients at subsensitive concentrations

**Figure 6:**
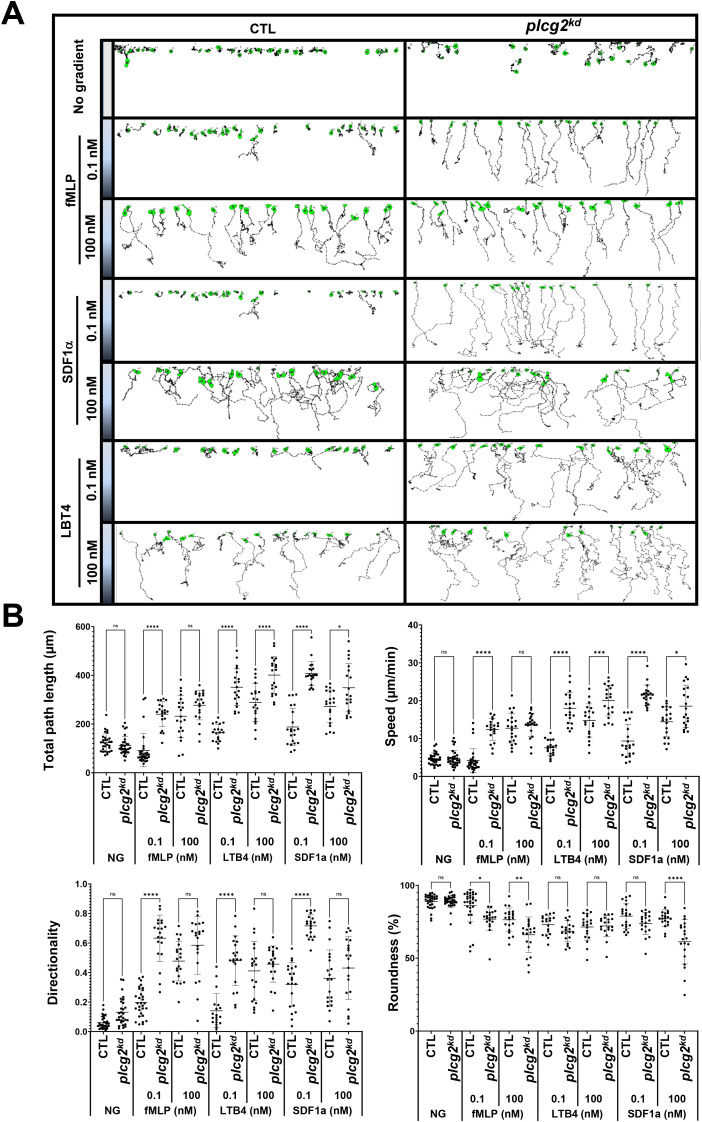
*plcg2^kd^* neutrophils display improved chemotaxis in chemoattractant gradients at subsensitive concentrations. **(A)** Montages show the travel path of chemotaxing CTL or *plcg2^kd^* cells in response to subsensitive or mid-concentration gradients. The plain or shaded panels on the left side of the images in the montage indicate either no gradient (NG) or chemoattractant gradients of fMLP (top), SDF1a (middle), or LTB4 (bottom) sourced from the indicated concentrations. The concentration on the top side of the terrace is 0 and the concentration at the bottom side of the terrace is as indicated on the left side of the terrace. Movement of at least 30 cells in each group was analyzed by DIAS software and is shown. See **Supplementary Video S12** (CTL, left, and *plcg2^kd^*, right) for a complete set of ez-taxiscan images with the same conditions of chemoattractant concentrations shown in **A. (B)** Chemotaxis behaviors measured from A are described as four parameters: directionality, which is “upward” directionality, where 0 represents random movement and 1 represents straight movement toward the gradient; speed, defined as the distance that the centroid of the cell moves as a function of time; total path length, the total distance the cell has traveled; and roundness (%) for polarization, which is calculated as the ratio of the width to the length of the cell. Thus, a circle (no polarization) is 1, and a line (perfect polarization) is 0. Thirty cells from each group were measured for 10 min. Mean ± SD are shown. A student’s *t*-test was used to calculate the *p*-values. Statistical significance is indicated as follows: *ns* (not significant *p* > 0.05), *(*p* < 0.0.05), **(*p* < 0.01), ***(*p* < 0.001), or ****(*p* < 0.0001).

We found that *capri^kd^* neutrophils, which lack Ras inhibitor CAPRI, display an increased sensitivity and elevated activation of Ras and its downstream effectors (23). More importantly, *capri^kd^* neutrophils display an altered chemotaxis behavior: an improved chemotaxis in the gradients at subsensitive concentrations, a normal chemotaxis in the gradients at medium concentrations, and an impaired chemotaxis in the gradients at saturating concentrations. That is, neutrophils lacking CAPRI display an upshift in concentration range for chemotaxis (30). We have previously shown an impaired chemotaxis of *plcg2^kd^* cells upon chemoattractant gradients at a saturating concentration (20). Next, we examined the chemotaxis behavior of CTL and *plcg2^kd^* cells in the gradients of three chemoattractants at either medium (100 nM) or subsensitive (0.1 nM) concentrations (**Figure 6**, **Supplementary Video S12**). In the absence of a gradient, *plcg2kd* cells appeared to exhibit a broader random walk compared to CTL cells, although the difference was not statistically significant (**Figures 6A, B**). When exposed to gradients generated from a source at medium concentrations (100 nM), CTL and *plcg2^kd^* cells displayed overall similar chemotaxis capability, although *plcg2^kd^* cells displayed slightly decreased speed and total path length (**Figure 6B**). In the gradients generated from the sources of 0.1 nM, most CTL cells displayed random migration, while most *plcg2^kd^* cells displayed a clear directed cell migration along the direction of the gradient. In conclusion, *plcg2^kd^* cells display a concentration-dependent, altered chemotaxis behavior: an improved chemotaxis in the gradients at a subsensitive concentration, while a normal chemotaxis in the gradients at medium concentrations. Combined with the previous report (20), our results demonstrate that neutrophils lacking PLCγ2 display an upshift of the concentration ranges of diverse chemoattractants for an efficient chemotaxis.

## Discussion

**Figure 7:**
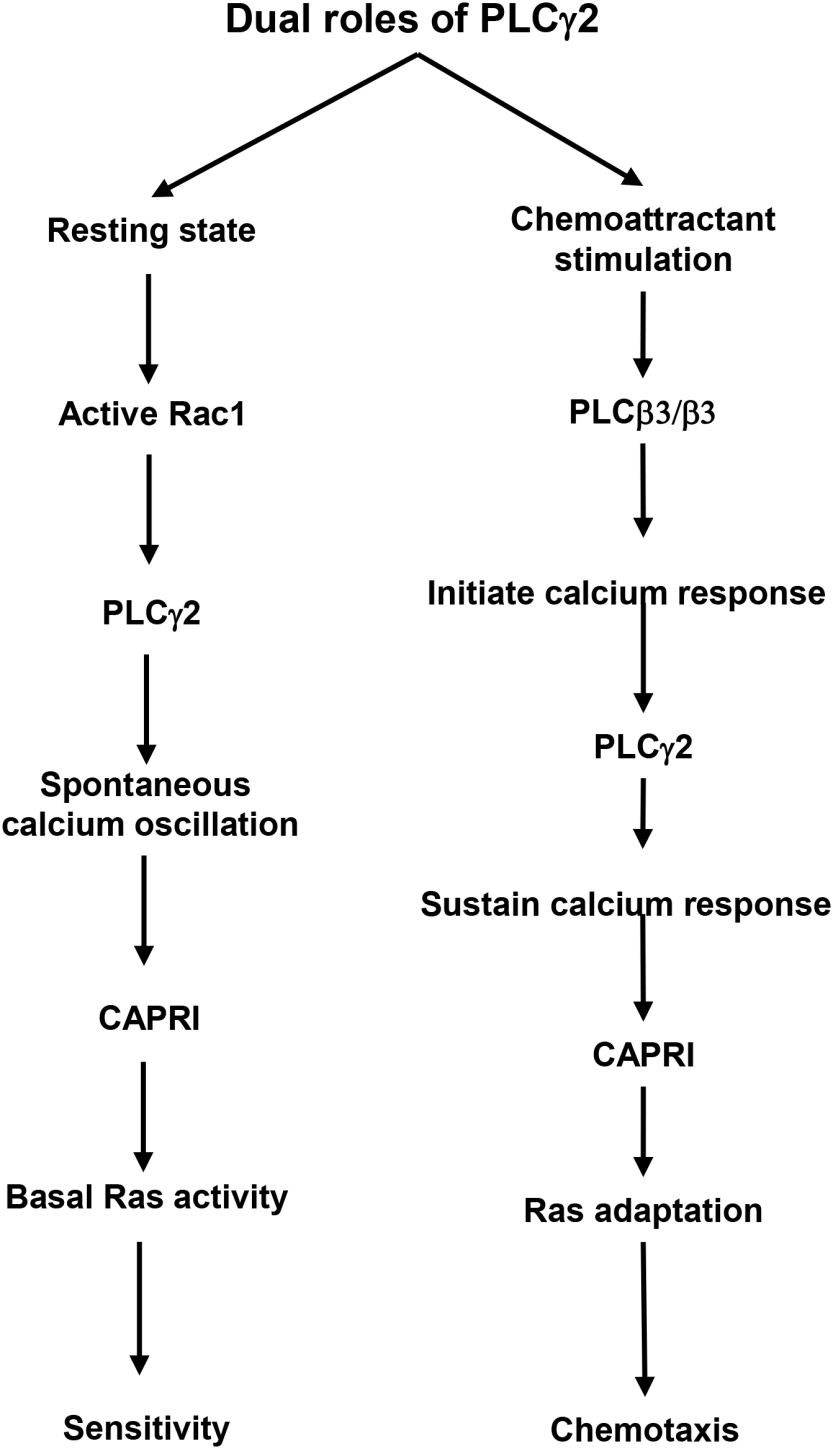
A schematic illustration of the dual roles of PLCγ2 in controlling cell sensitivity and GPCR-mediated chemotaxis.

Chemoattractant-triggered PLCβ2/β3 activation and the essential role of PLCβ2/β3 in subsequent calcium signaling in neutrophils have been previously characterized (2). However, no connection has been made between calcium oscillation and cell sensitivity toward chemoattractants. The mediator(s) or biological functions of calcium oscillation in neutrophils largely remain elusive. In the present study, we show that PLCγ2-mediated spontaneous calcium oscillation controls the basal Ras activity and neutrophil sensitivity by the recruitment of CAPRI to the plasma membrane. More importantly, the chemoattractant-induced PLCγ2 activation constitutes the essential calcium response for the PM recruitment of CAPRI and subsequent adaptation of Ras and downstream effectors for proper chemotaxis. Hence, by applying the above two mechanisms, PLCγ2 gates the chemoattractant concentration range for neutrophil chemotaxis (**Figure 7**).

Calcium oscillation is ubiquitous, triggered either spontaneously or upon receptor-ligand binding in all cells. The PLC-derived, IP_3_-mediated intracellular Ca^2+^ release triggers the initial [Ca^2+^] increase and constitutes calcium oscillation and calcium influx, which includes the entry of Ca^2+^ through the activation of store-operated channels (SOCs) in the plasma membrane. Murine PLCβ2/β3-deficient (*plcb2^-/-^b3^-/-^*) neutrophils display a significant decrease in IP_3_ production and calcium response, demonstrating the essential role of PLCβ2/β3 in chemoattractant-mediated calcium response (2). However, mammalian neutrophils express three main isoforms of PLC, including -β2, -β3, and -γ2 (15). We previously reported that chemoattractant stimulation induces robust plasma membrane translocation of PLCγ2 (17), which is a highly expressed PLC isoform that can be activated by membrane translocation (18, 19). We also found that the membrane translocation of PLCγ2 requires its C2-domain (20). To delineate the contribution of PLCβ2/β3 and γ2 in chemoattractant-induced calcium signaling, we monitored calcium response in *plcg2^kd^* cells, which express endogenous PLCβ2/β3. In these cells, the chemoattractant stimulation-triggered calcium response results from the activation of PLCβ2/β3. The observed difference between CTL and *plcg2^kd^* cells is the contribution of PLCγ2 in this process. *plcg2^kd^* neutrophils display a concentration-dependent calcium response: in response to stimuli at a saturating dose, *plcg2^kd^* neutrophils display calcium responses with a normal amplitude, but with significantly decreased duration or secondary (oscillatory) calcium response (20); upon medium (10 nM fMLP) or low (1 nM fMLP) stimuli, *plcg2^kd^* neutrophils display significantly reduced calcium response in both amplitude and duration; upon subsensitive stimuli, *plcg2^kd^* neutrophils do not display spontaneous calcium oscillation (**Figure 1**). Taken together, the above results indicate that PLCβ2/β3 is responsible for the initial calcium response upon chemoattractant stimulation, and PLCγ2 is responsible for sustaining the calcium response and mediating the spontaneous calcium oscillation.

Few connections have been made between calcium oscillation and cell sensitivity to extracellular stimuli. No clear biological function of calcium oscillation had been implicated in chemotaxis of neutrophils. In both the model organism *Dictyostelium* and mammalian neutrophils, Ras plays a central role in the signaling pathways of chemotaxis of eukaryotic cells and serves as a hallmark of basal cell sensitivity. Cells lacking negative regulators of Ras signaling, such as *c2gapA^-^ Dictyostelium* cells or *capri^kd^* neutrophils, often display an increased basal Ras activity and cell migration, and hypersensitivity to stimuli (30). The consequence is an upshift in the concentration range of chemoattractant gradients, in which cells can sense and chemotax. Specifically, these cells are able to sense and chemotax in gradient at subsensitive concentrations but fail to migrate effectively in the gradients at saturating concentrations. Plasma membrane (PM) targeting of these Ras GAPs is required for their functions and often depends on calcium signaling. In neutrophils, we found that the recruitment of CAPRI to PM is significant reduced in *plcg2^kd^* neutrophils in both resting and chemoattractant-stimulated conditions (**Figure 2**) (20). Similar to *capri^kd^* neutrophils, *plcg2^kd^* neutrophils also display an increased Ras activity, sensitivity, and an upshift in the concentration range of chemoattractant gradients (**Figures 2**–**6**) (20). Additionally, *plcg2^kd^* cells show slightly increased random migration, although we did not quantify chemokinesis in this context. Our result reveals a molecular mechanism by which neutrophils use PLCγ2-mediated spontaneous calcium oscillation to regulate cellular sensitivity to external stimuli. Moreover, PLCγ2-dependent calcium signaling is required to recruit CAPRI to the PM for Ras deactivation, enabling proper Ras adaptation (20). In conclusion, PLCγ2 serves as a critical regulator that gates the effective concentration range for neutrophil chemotaxis.

Although clinical reports on neutrophil function in patients with PLCγ2 deficiency are sparse, studies in *plcγ2^-/-^* mouse models offer translational relevance. In the brain, PLCγ2 is primarily expressed by microglia and loss of PLCγ2 function has subtle effects on brain homeostasis that may underlie enhanced vulnerability to AD pathology via microglia and myelin dysfunction (11). Two independent studies have reported that *plcγ2^-/-^* mice are protected from developing arthritis, suggeting a pro-inflammatory role for PLCγ2 in this context (16, 31). Consistent with our findings in HL60, *plcγ2^-/-^* mouse neutrophils exhibit improved chemotaxis in gradients generated from 100 nM fMLP, but impaired chemotaxis in gradients from a 300 nM fMLP source (16). Similarly, these two reports show that *plcγ2^-/-^* mouse neutrophils display increased cell migration, improved chemotaxis, compared to wild-type counterparts in *in vitro* chemotaxis assays done with chemoattractants at low concentration assay or *in vivo* chemotaxis assays (16, 31). These findings are particularly relevant given that circulating and tissue-local chemoattractant concentrations in physiological and inflammatory contexts are often low (32–34). In agreement with these observations, we found that *plcg2^kd^* human neutrophil-like cells display concentration-dependent chemotaxis: they show improved chemotaxis in gradients of multiple chemoattractants at low or subsensitive concentrations, but impaired chemotaxis in gradients at saturating concentrations (**Figure 6**) (20). This suggests a conserved role of PLCγ2 in modulating neutrophil sensitivity and chemoattractant concentration-range detection. Future work using primary human neutrophils or patient-derived samples will be essential to validate these mechanisms and further understand their relevance to immune regulation and disease pathogenesis.

## Data Availability

The original contributions presented in the study are included in the article/**Supplementary Material**. Further inquiries can be directed to the corresponding author.
